# Post-COVID-19 vaccine medium-vessel vasculitis and acute anterior uveitis, causation vs temporal relation; case report and literature review

**DOI:** 10.1016/j.amsu.2022.103407

**Published:** 2022-02-24

**Authors:** Abdul-Wahab Al-Allaf, Almurtada Razok, Yousr Al-Allaf, Loai Aker

**Affiliations:** a Department of Rheumatology, Hamad Medical Corporation, P.O 3050, Doha, Qatar; b Department of Internal Medicine, Hamad Medical Corporation, P.O 3050, Doha, Qatar; c Imperial College School of Medicine, London, United Kingdom; d Department of Radiology, Hamad Medical Corporation, P.O 3050, Doha, Qatar

**Keywords:** COVID-19 vaccine, Vasculitis, Celiac trunk, Anterior uveitis, Immunosuppressive medications

## Abstract

**Introduction:**

and importance: Multiple immunologic phenomena were reported following the administration of COVID-19 vaccines. However, the important point is that their possible association with medium-vessel vasculitis involving the celiac trunk and its branches with acute anterior uveitis in the same patient has not been reported before.

**Case presentation:**

In this manuscript, we are reporting a case of a middle-aged gentleman who developed vasculitis involving the celiac trunk and its branches, and acute anterior uveitis one week and three weeks after the second dose of Pfizer BioNTech COVID-19 vaccine, respectively. The patient showed significant clinical and radiographic improvement after receiving corticosteroids and azathioprine.

**Clinical discussion:**

Previously reported cases of vasculitis following COVID-19 vaccines included both renal-limited and more generalized vasculitis with some being positive and others negative for ANCA (anti-neutrophil cytoplasmic antibodies). Nevertheless, it is worth mentioning that most cases responded to immunosuppressive treatment. Post-COVID-19 vaccine uveitis was reported in patients with different age spans including both anterior and posterior uveitis, with remission being achieved after the use of corticosteroids.

**Conclusions:**

Multiple cases of vasculitis and acute anterior uveitis were reported following COVID-19 vaccines; however, it is important to mention that more research is needed to establish an association between the COVID-19 vaccine and both vasculitis and acute anterior uveitis. In our opinion, the benefits of the COIVID-19 vaccine largely outweigh the expected risks.

## Highlights

• This is the first case report for medium vessel vasculitis after a COVID-19 vaccine.
• The association could be casual or by chance. More studies are needed to assert the nature of this association.
• We think that this is a transient condition and it could be managed in the same line for managing vasculitis.
• This should not deter us from using the COVID vaccine, which is essential to alter the course of the COVID epidemic.

## 1 Introduction

Since the emergence of the COVID-19 virus in late 2019, the world has been preoccupied with its negative impacts on public health worldwide. Due to the catastrophic effects of the COVID-19 pandemic, the Food and Drug Administration (FDA) approved the use of multiple vaccines on an emergency basis, which include Pfizer BioNTech, Moderna and Johnson & Johnson [1]. After the widespread use of COVID-19 vaccines, a variety of local and systemic reactions were observed. The local side effects included pain, erythema and swelling [2], but more worrying were the various systemic reactions including a variety of immune-mediated conditions such as Guillain-Barre syndrome, Vaccine‐associated Immune Thrombosis and Thrombocytopenia (VITT) syndrome and myocarditis [3]. Many cases of small-vasculitis following the COVID-19 vaccine were reported [4,5]. We report a case of isolated medium-vessel vasculitis involving the celiac trunk and its branches one week after the second dose of the Pfizer BioNTech COVID-19 vaccine. To the best of our knowledge, this is the first case report of post-COVID-19 vaccine celiac trunk vasculitis. We would like our physician colleagues to be vigilant about such an association. However, at the same time, we would like to emphasize that the benefits of the COVID-19 vaccine outweigh these possible associated adverse reactions and that such temporal relation is not approved for causation. More research is needed to establish a causal relationship between these reactions and COVID-19 vaccines.

## 2 Presentation of case

**Fig. 1:**
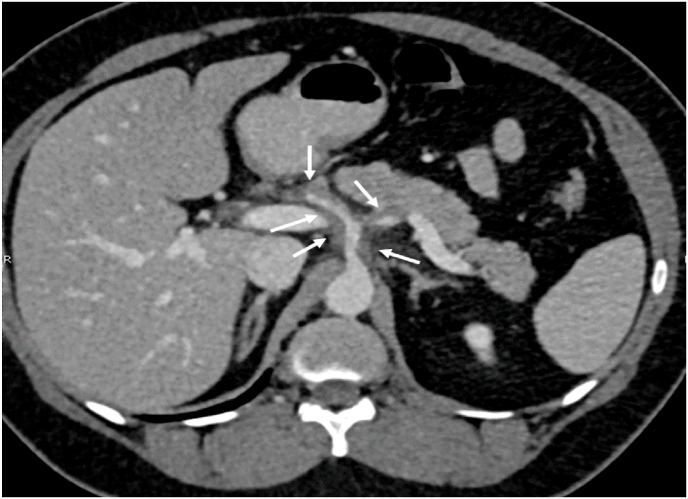
Axial contrast-enhanced CT image acquired at the level of the celiac artery showing circumferential thickening of the wall of the celiac, splenic, and common hepatic arteries (white arrows) with mural enhancement indicating inflammation.

A 46-year-old gentleman who is overweight, smoker, with no history of alcohol consumption or any significant family history, with the background of well-controlled essential hypertension on amlodipine 5 mg daily. He presented to the emergency department (ED) on March 16, 2021 with a chief complaint of epigastric and left upper quadrant abdominal pain for six days duration. The pain was non-radiating, severe in intensity, rated as 7/10 as per the normalized scale ratio (NRS), stabbing in nature and did not respond adequately to oral analgesics such as paracetamol and ibuprofen. There was no nausea, vomiting, alteration in bowel habits or urinary complaints. He had received the second dose of the Pfizer BioNTech COVID-19 vaccine a week before that, on March 09, 2021, after which he experienced fever and rigors for the first day. Both doses of the vaccine were administered in the left deltoid muscle area. He worked as a driver and had not recently travelled. The history was negative for photosensitivity, oral ulcers, joint pain, sicca symptoms, lymphadenopathy, weight loss and night sweats. In the ED, he had normal vital signs including oral and axillary temperature. His labs were pertinent for mildly elevated C-reactive protein of 20.3 mg/dl (reference range 0–5 mg/dl) but were otherwise normal including negative serum lipase and amylase. Erythrocyte sedimentation rate (ESR) was 11 mm/hr (reference range 2–28 mm/hr). Serologic autoimmune and vasculitis panels were negative, including ANA, anti-DS-DNA, Anti-RO, anti-LA, anti-SCl-70, anti-phospholipid screening, and ANCA with normal C3 and C4. Computed tomography (CT) scan of the abdomen with contrast revealed a fat stranding along the celiac trunk, splenic, and common hepatic arteries with circumferential thickened vascular walls with mural enhancement indicating inflammation suggestive of focal vasculitis (Fig. 1).

**Fig. 2:**
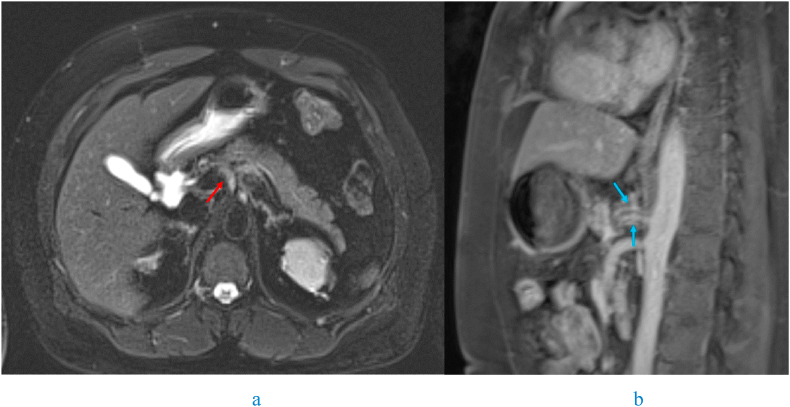
a and bFat-saturated axial T2-weighted image showing the increased signal intensity of the celiac artery wall (red arrow) compatible with oedema and inflammation (figure a). Sagittal T1-weighted fat-saturated gadolinium-enhanced MR image showing circumferential thickening and increased mural enhancement of the wall of the celiac artery (blue arrows) in addition to luminal narrowing (figure b).

Furthermore, confirmation using magnetic resonance imaging of the abdominal vessels wall (MRA) was obtained and revealed diffuse circumferential wall thickening with luminal narrowing and irregularity of the celiac trunk, splenic, hepatic, and left gastric arteries (Fig. 2a and b).

**Fig. 3:**
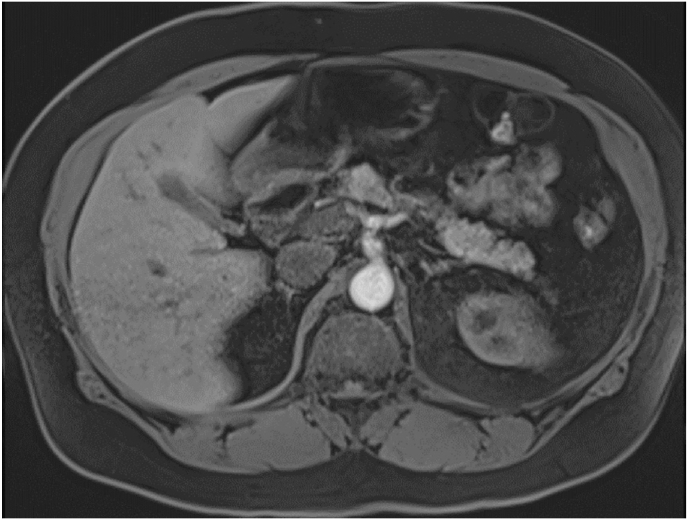
Follow up axial T1-weighted fat-saturated gadolinium-enhanced MR image showing regression of the mural and perivascular thickening previously seen in the celiac artery and its branches with the restoration of the lumen suggesting improvement of the underlying inflammatory process.

The aorta, superior and inferior mesenteric, renal, and iliac arteries were uninvolved. Based on the aforementioned findings, he was diagnosed with medium-vessel vasculitis and was started on intravenous methylprednisolone 250 mg once daily for three days, after which he had a significant improvement in his abdominal pain. He was discharged home on a reducing dose of oral prednisolone 60 mg once daily with a plan for gradual tapering by 5 mg every week. As he is prediabetic, he has been started on Metformin 500 mg bid. Three weeks after he received the second dose of the Pfizer BioNTech COVID-19, the patient developed acute-onset pain, erythema, photophobia and blurring of vision in his right eye. He was seen urgently by an ophthalmologist and a full and detailed examination confirmed the presence of acute anterior uveitis. He was started on topical triamcinolone drops. At the same time azathioprine, 50 mg once daily was initiated as a steroid-sparing agent, with a plan to increase the dose by 50 mg every two weeks until the dose of 150 mg daily which has been reached, as his blood tests including FBC and LFTs continued to be satisfactory. The patient was reviewed in the rheumatology and ophthalmology clinics three weeks later and there was complete resolution of the uveitis, in addition, there was no recurrence of abdominal pain. He managed to stop smoking on our advice. The repeat MRA five months later showed a reduction in the previously documented soft tissue thickening around the celiac trunk and its branches, with minimal thin residual rim (Fig. 3).

Furthermore, there was a resolution of the previously noted narrowing of the celiac trunk and its branches. Accordingly, after that, his steroid was completely stopped on September 28, 2021, around 6 months after his initial presentation and has been advised to continue with the Azathioprine 150 mg daily. He has been reviewed 3 months later and he continued to be in remission. The patient confirmed daily adherence to the prescribed treatment and did not report any therapy-related adverse events during the follow-up period.

**Patient perspective:** “Initially, I was really worried after I was told I had inflammation in both my abdomen and eye, and I kept thinking whether the two events were inter-related or related to the COIVD-19 vaccine, I guess only time will tell”.

This case report has been reported in line with the SCARE 2020 Guideline criteria [6].

## 3 Discussion

New-onset vasculitis following COVID-19 vaccination was reported in multiple patients. Of note, two case reports described renal-limited, ANCA-positive vasculitis in patients who were found to have acute kidney injury. The first case was about a 78-year-old lady who presented with nausea and vomiting 16 days after receiving the Pfizer-BioNTech COVID-19 vaccine and was found to have antibodies against myeloperoxidase (MPO) and pauci-immune necrotizing glomerulonephritis. Renal function parameters improved after receiving corticosteroids and rituximab [7]. The second case reported, was a 52-year-old gentleman who presented with headache and weakness two weeks after receiving the second dose of the Moderna mRNA vaccine and was also found to have pauci-immune necrotizing glomerulonephritis but with antibodies against proteinase-3 (PR3). Unfortunately, his renal function deteriorated despite administering rituximab and cyclophosphamide, which necessitated the initiation of hemodialysis [8].

An additional case report described a 77-year-old gentleman who presented with fever and night sweats four weeks following the first dose of the AstraZeneca COVID-19 vaccine and was found to have acute non-necrotizing granulomatous nephritis which subsided within four weeks of receiving methylprednisolone. Interestingly, the patient had negative anti-neutrophil cytoplasmic antibodies, however, there was evidence of medium-vessel involvement on positron emission tomography scan (PET) [9].

In relation to post-COVID-19 vaccine uveitis, multiple cases were reported as well, and both anterior and posterior uveitis were observed. In one report, a 15-year-old girl with a background of antinuclear antibodies-positive juvenile idiopathic arthritis developed bilateral anterior uveitis five days after receiving the second dose of the Sinopharm COVID-19 vaccine. Her condition improved after receiving topical steroids [10]. In addition, a case of bilateral posterior uveitis was reported in a 50-year-old lady with no medical background who developed bilateral blurred vision also five days after receiving the inactivated COVID-19 vaccine. The patient responded to therapy with topical and systemic steroids [11].

The unique and novel aspects of the case we are reporting include the post-COVID-19 vaccine medium-vessel vasculitis involving the celiac trunk and its branches and the development of unilateral acute anterior uveitis, in addition to the co-existence of both conditions in the same patient, which to the best of our knowledge, has not been reported before.

Considering the inability to confirm the association between vasculitis and uveitis on one hand and the COVID-19 vaccine on the other on the molecular level, despite the temporal association, we cannot presume for sure that the vaccine is the causative agent for his vasculitis and the uveitis.

We would like to express our opinion that the benefits of the COVID-19 vaccine outweigh the possible associated risks on both the individual and community levels and that more research is needed to confirm or rule out the possible association between the COVID-19 vaccine and the adverse events reported in this and other manuscripts.

## 4 Conclusion and learning points

Possible complications of COVID-19 vaccines have become of special interest to both healthcare workers and the public. We report this case to raise awareness about abdominal vasculitis and acute anterior uveitis as possible complications of COVID-19 vaccination. We hope that our manuscript will serve as a bridge for future studies that will further investigate these topics and will alarm healthcare workers to consider vasculitis as a cause of otherwise unexplained abdominal pain following the COVID-19 vaccine.

This work was reported in line with the SCARE guidelines [6].

## Availability of data and materials

The datasets used and/or analyzed during the current study are available from the corresponding author on reasonable request.

## Ethical approval

Written informed consent was obtained from the patient for publication of this case report and the accompanying images. A copy of the written consent is available for review by the Editor-in-Chief of this journal on request.

## Source of funding

NA/this article did not receive any specific grant from funding agencies in the public, commercial, or not-for-profit sectors.

## Author contribution statement

AWA provided the case who is under his care and provided advisory and scientific support, he reviewed and proved the final version. AR performed the literature review and wrote the first draft of the manuscript. YA reviewed and edited the manuscript from a scientific and English language point of view to its final version. LA provided informative radiological images for the case and their captions.

## Consent for publication

Written informed consent was obtained from the patient for publication of this case report and the accompanying images. A copy of the written consent is available for review by the Editor-in-Chief of this journal on request.

## Registration of research studies

Not applicable, the manuscript is a case report.

## Research registration

N/A.

## Provenance and peer review

Not commissioned, externally peer-reviewed.

## Guarantor

Abdul-Wahab Al-Allaf.

## Declaration of competing interest

None to be declared.
